# GSTM1 Modulates Expression of Endothelial Adhesion Molecules in Uremic Milieu

**DOI:** 10.1155/2021/6678924

**Published:** 2021-01-25

**Authors:** Djurdja Jerotic, Sonja Suvakov, Marija Matic, Abdelrahim Alqudah, David J. Grieve, Marija Pljesa-Ercegovac, Ana Savic-Radojevic, Tatjana Damjanovic, Nada Dimkovic, Lana McClements, Tatjana Simic

**Affiliations:** ^1^Institute of Medical and Clinical Biochemistry, Faculty of Medicine, University of Belgrade, 11000 Belgrade, Serbia; ^2^Faculty of Medicine, University of Belgrade, 11000 Belgrade, Serbia; ^3^Division of Nephrology and Hypertension, Mayo Clinic, Rochester, MN, USA; ^4^Department of Clinical Pharmacy and Pharmacy Practice, Faculty of Pharmaceutical Sciences, The Hashemite University, P.O. Box 330127 Zarqa 13133, Jordan; ^5^The Wellcome-Wolfson Institute for Experimental Medicine, School of Medicine, Dentistry and Biomedical Sciences, Queen's University Belfast, Belfast, UK; ^6^Clinical Department for Renal Diseases, Zvezdara University Medical Center, 11000 Belgrade, Serbia; ^7^School of Life Sciences, Faculty of Science, University of Technology Sydney, 2007, NSW, Australia; ^8^Serbian Academy of Sciences and Arts, 11000 Belgrade, Serbia

## Abstract

Deletion polymorphism of glutathione S-transferase M1 (GSTM1), a phase II detoxification and antioxidant enzyme, increases susceptibility to end-stage renal disease (ESRD) as well as the development of cardiovascular diseases (CVD) among ESRD patients and leads to their shorter cardiovascular survival. The mechanisms by which GSTM1 downregulation contributes to oxidative stress and inflammation in endothelial cells in uremic conditions have not been investigated so far. Therefore, the aim of the present study was to elucidate the effects of GSTM1 knockdown on oxidative stress and expression of a panel of inflammatory markers in human umbilical vein endothelial cells (HUVECs) exposed to uremic serum. Additionally, we aimed to discern whether *GSTM1-null* genotype is associated with serum levels of adhesion molecules in ESRD patients. HUVECs treated with uremic serum exhibited impaired redox balance characterized by enhanced lipid peroxidation and decreased antioxidant enzyme activities, independently of the GSTM1 knockdown. In response to uremic injury, HUVECs exhibited alteration in the expression of a series of inflammatory cytokines including retinol-binding protein 4 (RBP4), regulated on activation, normal T cell expressed and secreted (RANTES), C-reactive protein (CRP), angiogenin, dickkopf-1 (Dkk-1), and platelet factor 4 (PF4). GSTM1 knockdown in HUVECs showed upregulation of monocyte chemoattractant protein-1 (MCP-1), a cytokine involved in the regulation of monocyte migration and adhesion. These cells also have shown upregulated intracellular and vascular cell adhesion molecules (ICAM-1 and VCAM-1). In accordance with these findings, the levels of serum ICAM-1 and VCAM-1 (sICAM-1 and sVCAM-1) were increased in ESRD patients lacking GSTM1, in comparison with patients with the *GSTM1-active* genotype. Based on these results, it may be concluded that incubation of endothelial cells in uremic serum induces redox imbalance accompanied with altered expression of a series of cytokines involved in arteriosclerosis and atherosclerosis. The association of GSTM1 downregulation with the altered expression of adhesion molecules might be at least partly responsible for the increased susceptibility of ESRD patients to CVD.

## 1. Introduction

Endothelial dysfunction is an underlying mechanism of cardiovascular diseases (CVD) which are the leading cause of death among patients with end-stage renal disease (ESRD) [1–4]. The vascular endothelium is likely the primary target of uremic toxins generated in ESRD. In these conditions, the endothelium is continuously exposed to accumulated uremic toxins hence inducing oxidative stress and inflammation, which can lead to endothelial impairment [2, 5–7].

Homozygous deletion of glutathione S-transferase M1 (GSTM1), which is a phase II detoxification enzyme, leads to accumulation of oxidative stress byproducts which indicates its role in antioxidant protection as well [8]. Between 30 to 50 percent of different human population are homozygous for GSTM1 deletion (usually denoted as *GSTM1-null* genotype), thus completely lacking the GSTM1 enzyme [9]. The GSTM1 deficiency is linked to higher susceptibility to CVD as individuals with *GSTM1-null* genotype were shown to have significantly increased risk for developing resistant hypertension [10], coronary artery disease/atherosclerosis [11, 12], and stroke [13, 14]. In addition, the *GSTM1-null* genotype increases susceptibility to ESRD and leads to shorter overall and cardiovascular survival of the patients on haemodialysis [8, 15–17]. The association between GSTM1 deletion and oxidative stress in ESRD patients was supported by our previous results that demonstrated elevated levels of several byproducts of protein and lipid oxidative damage in ESRD patients with *GSTM1-null* genotype compared to those with *GSTM1-active* genotype [8]. It has been suggested that in inflammatory and prooxidant environment observed in patients with ESRD, the endothelium responds by expressing intracellular and vascular cell adhesion molecules (ICAM-1 and VCAM-1) that facilitate migration and adhesion of leukocytes to the endothelial cells [18]. We have recently shown that soluble ICAM-1 and VCAM-1 (sICAM-1 and sVCAM-1) levels have a strong predictive role in terms of overall and cardiovascular survival in ESRD patients on haemodialysis [17]. Nevertheless, the potential influence of GSTM1 deletion on the aforementioned inflammatory markers has not been elucidated before in dialyzed patients or in endothelial cells exposed to uremic toxins commonly present in ESRD.

Despite the convincing findings in human cohorts showing the importance of GSTM1 deletion on susceptibility and development of CVD among dialyzed patients [15, 16, 19, 20], its role in the vascular pathophysiology has not been established yet. A functional role of GSTM1 was investigated in vascular smooth muscle cells (VSMC), showing that the reduction in GSTM1 expression in these cells caused increased cell proliferation, oxidative stress, and migration [21]. However, the mechanisms associated with GSTM1 downregulation in endothelial cells in uremic conditions have not been investigated so far.

Therefore, the aim of the present study was to elucidate the effects of GSTM1 knockdown on oxidative stress and expression of a panel of inflammatory markers in human umbilical vein endothelial cells (HUVECs) exposed to uremic serum. Additionally, we aimed to discern whether the *GSTM1-null* genotype is associated with serum levels of adhesion molecules in ESRD patients.

## 2. Materials and Methods

### 2.1. Cell Cultures

HUVECs (*ATCC Manassas*, *Virginia*, *USA*) were seeded on 0.2% gelatine-coated culture plates and grown in a MV2 growth medium (*Endothelial Cell Growth Medium MV2*, *PromoCell*, *Germany*) under standard cell culture conditions (humidified atmosphere, 5% CO_2_, 37°C). Cells were seeded in gelatine-coated plates for viability assays, Western blot analyses, oxidative stress measurements, and assessment of cytokine expression. HUVECs were incubated in a pool of human serums obtained from either healthy volunteers (control serum, *n* = 10) or ESRD patients on haemodialysis (uremic serum, *n* = 30). All patients underwent haemodialysis 3 times a week for at least 3 months before the study onset using polysulfone dialysis membranes and conventional bicarbonate-buffered dialysate. Participants with any form of malignancy, autoimmune disease, or infectious comorbidities (HIV, HBV, or HCV infections) were excluded. The blood was taken from patients prior to a haemodialysis session at the Center for the Renal Diseases, Zvezdara University Medical Center, Belgrade. All patients signed informed consent agreeing to participate in the study. This study was approved by the Ethical Committee of the Faculty of Medicine, University of Belgrade (No. 1550/V-30), and conducted in accordance with the Helsinki Declaration from 2013.

### 2.2. Cell Viability Assay

**Figure 1:**
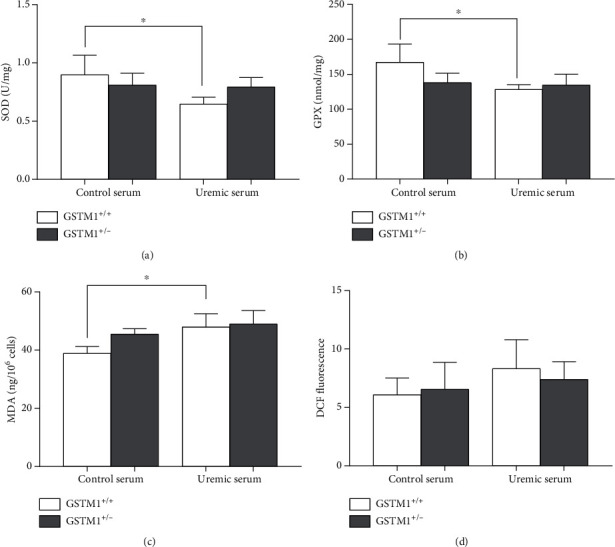
The influence of uremic serum and GSTM1 knockdown on oxidative stress parameters in HUVECs. GSTM1^+/+^ and GSTM1^+/-^ HUVECs were incubated in 30% control and 30% uremic serum-containing media for 6 h. (a) SOD activity and (b) GPX activity were determined by spectrophotometry. (c) The total ROS were determined by flow cytometry after DCF staining. (d) MDA levels were determined by ELISA. Results are presented as the mean ± SD, *n* = 3. ^∗^*p* < 0.05.

Cell viability was assessed by a colorimetric method based on measuring mitochondrial dehydrogenase activity, using the *MTS Cell Proliferation Assay Kit* (*Abcam, UK*), according to the manufacturer's protocol. 5000 cells/well were cultured in a 96-well plate. After 24 h, growth media were discarded and cells were treated with media, 10%, 20%, or 30% control or uremic serum for 4 h and 6 h. The formazan salt produced by viable cells was quantified by measuring the absorbance at 490 nm on the *FLUOstar® Omega plate reader* (*BMG Labtech*, *Germany*). The viability of cells incubated in pooled human sera for 6 h did not change significantly compared to the cells incubated in the normal growth medium (Figure 1S A&B, Supplement). Therefore, all further HUVEC treatments were performed using 30% control or uremic serum-containing media for 6 h, before cytokine expression and oxidative stress measurements were performed.

### 2.3. GSTM1 Knockdown Using siRNA in HUVECs

To silence GSTM1 protein expression, HUVECs were seeded at a density of 1.5 × 10^5^ cells per well in 6-well plates and grown in a MV2 growth medium. The following day, cells were transfected with 100 nM GSTM1 small interfering RNA (siRNA) (*Thermo Fisher Scientific*, *UK*) using the DharmaFECT transfection reagent (*GE Healthcare*, *USA*) or treated with DharmaFECT as a control. Ninety-six hours posttransfection, the silencing effect was confirmed by Western blot.

### 2.4. Western Blot Analysis

HUVECs were lysed in a RIPA buffer (50 mM Tris-HCL pH 8.0, 150 mM NaCl, 1% IGEPAL, 0.5% sodium deoxycholate, and 10% SDS) supplemented with protease inhibitor cocktail (*Roche*, *UK*). After extraction, protein concentrations were determined by the *Bicinchoninic Acid Protein Assay kit* (*BCA*, *Thermo Fisher Scientific*, *UK*). An equal amount of proteins was loaded on 10% polyacrylamide gel, and electrophoresis was performed at 80 V for 10 min, then 100 V for 90 min. After wet transfer, nitrocellulose membranes were blocked in 5% nonfat milk (*Bio-Rad*, *UK*) for 1 h at room temperature and then incubated overnight at 4°C with primary antibodies: monoclonal mouse anti-GSTM1 (1 : 1000, *R&D Systems*, *USA*), monoclonal mouse anti-ICAM1 (1 : 200, *Santa Cruz*, *USA*), polyclonal goat anti-VCAM1 (1 : 200, *Santa Cruz*, *USA*), and monoclonal mouse anti-*β* actin (1 : 10 000, *Thermo Fisher Scientific*, *UK*). The following day, membranes were incubated with appropriate HRP-conjugated secondary antibodies: anti-mouse 1 : 5000 (*Abcam*, *UK*) and anti-goat 1 : 1000 (*RayBiotech*, *USA*) for 1 h at room temperature. *C*hemiluminescent bands were detected using the *West Femto Maximum sensitivity substrate* (*Thermo Fisher Scientific*, *UK*) on the *G-box* (*Kodak*, *UK*) *or ChemiDoc* (*BioRad*, *USA*). Densitometry analysis was performed using *ImageJ* software (*National Institutes of Health*, *Bethesda*, *USA*).

### 2.5. Measurement of the Activity of Antioxidant Enzymes in HUVECs

The activity of antioxidant enzymes, superoxide dismutase (SOD) and glutathione peroxidase (GPX), in cell lysates was determined by spectrophotometric methods. SOD activity was assessed as previously described by Misra and Fridovich [22]. This method is based on the ability of SOD to inhibit the autoxidation of adrenaline at pH 10.2. The production of colored adrenochrome in reaction mixtures with cell protein extracts (sample) or without them (control) was followed at 480 nm. Activity of SOD was expressed as the percentage of inhibition of adrenaline autoxidation. GPX activity was assessed as reported by Günzler et al. [23]. GPX activity was assayed by the subsequent oxidation of NADPH at 340 nm with t-butyl-hydroperoxide as the substrate. One unit of enzyme activity was expressed as nmol NADPH oxidized per minute, assuming 6.22 × 10^3^/l/mol/cm to be the molar absorbance of NADPH at 340 nm.

### 2.6. Measurement of Malondialdehyde Levels in HUVECs

Malondialdehyde (MDA) levels in cell lysates were measured using the competitive ELISA kit (*Elabscience*, *Wuhan*, *China*) in accordance with the manufacturer's instructions. In brief, 50 *μ*l of standards, samples, and blanks was added to each well of the MDA precoated ELISA plate with consecutive addition of 50 *μ*l biotinylated antibody. After the 45 min of incubation, wells were washed in order to eliminate excess conjugate and unbound sample or standard, and HRP-conjugated antibody was added. The color change was measured spectrophotometrically at a wavelength of 450 nm.

### 2.7. Measurement of Total Reactive Oxygen Species in HUVECs

The total reactive oxygen species (ROS) production was assessed by flow cytometry (FACS) using 2′,7′-dichlorodihydrofluorescein diacetate (H_2_DCFDA; *Invitrogen*, *California*, *USA*) stain. HUVECs were seeded in 6-well plates (1.5 × 10^5^ cells/well) and transfected with GSTM1 siRNA as described above. After 90 h incubation, the transfection solution was discarded, and cells were incubated for the next 6 h with the 30% control or uremic serum-containing media. Treatments were removed, and cells were trypsinised. Cell pellets were resuspended in 5 ml flow cytometry buffer (1% FBS in PBS) and incubated with 5 *μ*l H_2_DCFDA stain for 30 min at 37°C. FACS tubes were centrifuged at 400 g for 8 min, supernatants were removed, and cells were allowed to recover for 15 min at 37°C in 1 ml of MV2 growth media. In the final step, cells were resuspended in 500 *μ*l FACS buffer, and 5 *μ*l *7-AAD-viability staining solution* (*eBioscience*, *San Diego*, *USA*) was added prior to performing measurements on the *Attune NxT Acoustic Focusing Flow Cytometer* (*Invitrogen*, *California*, *USA*). The results were analysed using *FlowJo*, *ver. 10.4* (*Stanford Jr. University*, *USA*).

### 2.8. Analysis of Cytokine Expression in HUVECs

To explore the effect of uremic serum and GSTM1 silencing on endothelial cell inflammation, the relative expression of 105 cytokines was assessed simultaneously in cell protein extracts using the *Proteome Profiler™ Human XL Cytokine Array Kit* (*R&D Systems*, *UK*) according to the manufacturer's instruction. HUVECs were seeded in 6-well Petri dishes at a density of 1.5 × 10^5^ cells per well. Following the transfection, GSTM1^+/+^ and GSTM1^+/-^ cells were incubated in 30% control or uremic serum-containing media for 6 h. After the incubation time expired, treatments were removed and cells were rinsed with PBS. Cells were then scraped in a *lysis buffer 17* (*R&D Systems*, *UK*), supplemented with 10 *μ*g/ml aprotinin, 10 *μ*g/ml leupeptin, and 10 *μ*g/ml pepstatin. Cell lysates were obtained after centrifugation at 14 000 g for 5 min. Pooled cell lysates (*n* = 3/group) were probed on four separate nitrocellulose membranes. Each membrane contained capture and control antibodies spotted in duplicate, which allowed simultaneous measuring of 105 cytokine expressions. Chemiluminescent spots were visualised on the *G-box* (*Kodak*, *UK*). Results were quantified using *HLimage++ software* and normalized to the reference spots positioned at three of the corners of each blot (*Western Vision Software*, *US*).

### 2.9. Analysis of Circulating Adhesion Molecules in Plasma of ESRD Patients

Circulating adhesion molecules were determined in plasma of ESRD patients by enzyme immunoassays, according to the manufacturer's protocols. The description of a cohort of 199 ESRD patients involved in this study has been described in details previously [17]. sICAM-1 was assayed by commercial solid-phase sandwich ELISA (*Thermo Fisher Scientific*, *Waltham*, *Massachusetts*, *USA*). sVCAM-1 was determined by a solid-phase sandwich ELISA kit (*Novex, Life Technologies*). Absorbances were read at 450 nm on the *LKB 5060-006 Micro Plate Reader* (*Vienna*, *Austria*). sICAM concentrations were expressed as pg/ml, and sVCAM levels were expressed as ng/ml.

## 3. Statistical Analysis

Statistical analysis was performed using the SPSS version 17.0 statistical package (*IBM SPSS Statistics*). All analysed parameters were tested for normality of the data using the Shapiro-Wilk test. For normally distributed data, differences between the groups were evaluated by the independent sample *t*-test or one-way ANOVA with Fisher's least significant difference (LSD) post hoc. For non-normally distributed data, the Mann-Whitney or Kruskal-Wallis test was used. The results were considered statistically significant if *p* < 0.05.

## 4. Results

**Figure 2:**
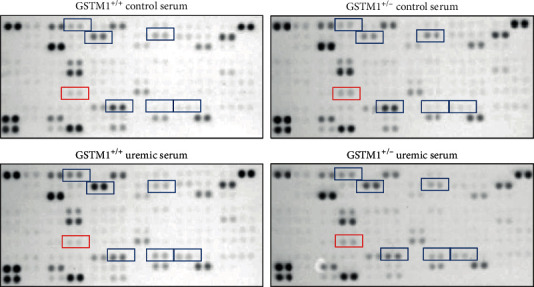
Proteome Profiler Human XL Cytokine Array and original blots. Cytokines that exhibited ≥2-fold change in uremic serum when compared to control serum treatment are marked blue (vertical comparison between images). Cytokines that exhibited ≥2-fold change in GSTM1^+/-^ cells when compared to GSTM1^+/+^ are marked red (horizontal comparison between images).

To determine the effects of GSTM1 expression on oxidative stress and expression of a panel of inflammatory markers, we used specific siRNA to silence GSTM1 gene in HUVECs. Following 96 h of transfection, diminished GSTM1 expression was confirmed by immunoblotting which showed around ~90% reduction in GSTM1 protein levels in HUVECs treated with GSTM1 siRNA (GSTM1^+/-^) compared to the control (GSTM1^+/+^) (*p* < 0.001; Figure 2S, Supplement).

### 4.1. The Influence of Uremic Serum and GSTM1 Knockdown on Biomarkers of Oxidative Stress (SOD, GPX, MDA, and ROS) in HUVECs

Antioxidant enzyme activity and byproducts of oxidative stress were assessed in GSTM1^+/+^ and GSTM1^+/-^ HUVECs incubated in control or uremic serum-containing media. The incubation of HUVECs with uremic serum led to a significant decrease in the activity of SOD and GPX antioxidant enzymes in GSTM^+/+^ HUVECs compared to control serum conditions (*p* < 0.05; Figures 1(a) and 1(b)). To determine the extent of GSTM1 activity loss on redox status of HUVECs, we silenced the GSTM1 gene by corresponding siRNA. Silencing of the GSTM1 gene did not affect antioxidant enzyme activity in any of the observed settings (Figures 1(a) and 1(b)). With respect to oxidative stress byproducts, the exposure to the uremic serum led to the significantly higher MDA concentrations (*p* < 0.05) in GSTM1^+/+^ HUVECs compared to their counterparts incubated in control serum (Figures 1(c) and 1(d)). However, the GSTM1 knockdown did not have statistically significant impact on total oxidative stress byproducts in HUVECs in either control or uremic serum (Figures 1(c) and 1(d)). Only a trend towards increased MDA levels was observed in GSTM1^+/-^ HUVECs compared to GSTM1^+/+^ HUVECs in control serum (*p* = 0.053).

### 4.2. The Influence of Uremic Serum and GSTM1 Knockdown on Cytokine Expression in HUVECs

In order to explore the effects of uremic serum and GSTM1 knockdown on endothelial cell inflammation, we assessed the relative expression of over 100 inflammatory markers with the proteome array. Pooled protein lysates (*n* = 3/group) from four different groups of treated cells (GSTM1^+/+^ control serum, GSTM1^+/-^ control serum, GSTM1^+/+^ uremic serum, and GSTM1^+/-^ uremic serum) were probed on four separate nitrocellulose membranes to measure cytokine expression. The array key and original blots are presented in Table 1 and Figure 2, respectively.

**Figure 3:**
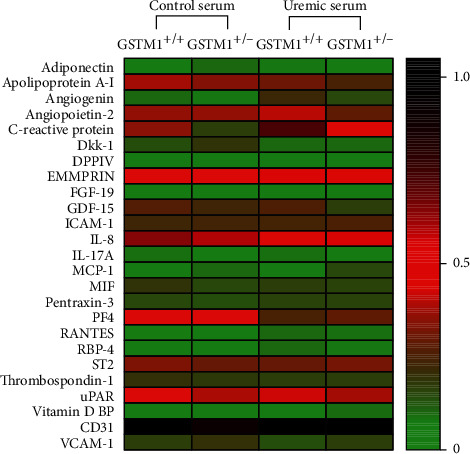
Expression of 25 cytokines determined by Proteome Profiler Human XL Cytokine Array. HUVECs (*n* = 3/group, pooled) transfected with GSTM1 siRNA and GSTM1^+/+^ HUVECs were incubated in 30% control or uremic serum-containing media for 6 h. Heatmap represents pixel densities of spots normalized by respective reference spots.

**Figure 4:**
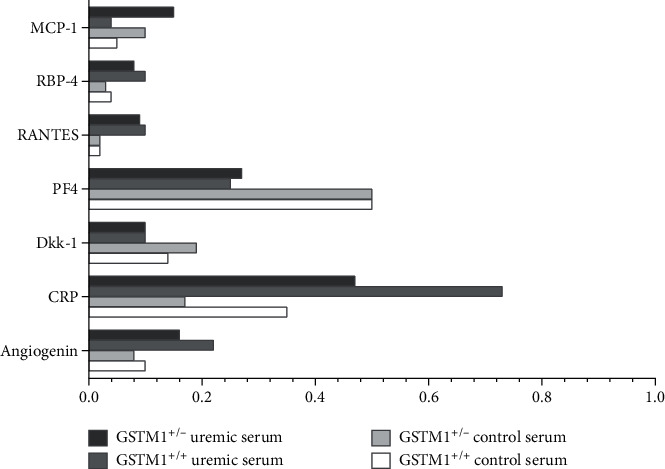
The relative expression of **c**ytokines that exhibited at least 2-fold change in response to GSTM1 knockdown or uremic serum treatment.

The relative expression of 103 cytokines is also presented as a graded heatmap (Figure 3S, Supplement) where the lowest expressions of proteins are showed in green, and the highest expression in black. The most highly expressed proteins in HUVECs, according to our results, are shown in Figure 3 in the manuscript. In addition, the proteins whose expression was changed 2-fold in response to GSTM1 knockdown or uremic serum treatment are presented as bars (Figure 4).

According to our analysis, the most highly expressed proteins in HUVECs were serpin, endoglin (data not shown due to their impact on the heatmap legend), and CD31. CD31 is well-established as one of the markers of HUVECs, which confirms the technical validity of the performed assay. The most prominent changes in cytokine expression were exerted by uremic serum treatment (Figures 2–4). Notably, incubation in uremic serum resulted in markedly higher expression of retinol-binding protein 4 (RBP4), regulated on activation, normal T cell expressed and secreted (RANTES), C-reactive protein (CRP), and angiogenin (Figure 4). Besides, the uremic serum treatment suppressed the expression of dickkopf-1 (Dkk-1) and platelet factor 4 (PF4). Interestingly, among numerous cytokines determined, the expression of only one protein was above the set threshold in response to GSTM1 knockdown. Namely, the monocyte chemoattractant protein-1 (MCP-1) expression increased 2-fold in GSTM1^+/-^ HUVECs incubated in control serum and 3.8-fold in GSTM1^+/-^ HUVECs incubated in uremic serum in comparison to corresponding GSTM1^+/+^ HUVECs.

### 4.3. The Influence of GSTM1 Deletion on ICAM-1 and VCAM-1 Expression in ESRD Patients and Endothelial Cells Exposed to Uremic Serum

**Figure 5:**
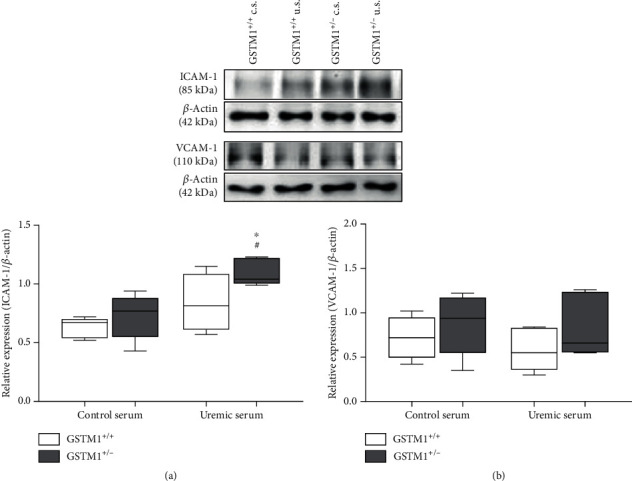
The influence of uremic serum and GSTM1 knockdown on ICAM-1 and VCAM-1 expression in HUVECs. GSTM1^+/+^ and GSTM1^+/-^ HUVECs were incubated in 30% control- (c.s.-) or 30% uremic serum- (u.s.-) containing media for 6 h. ICAM-1 and VCAM-1 expression was determined by Western blot. Results are presented as the median with interquartile range, *n* = 5. ^∗^*p* < 0.05 GSTM1^+/-^ HUVECs compared to GSTM1^+/+^ HUVECs; ^#^*p* < 0.05 GSTM1^+/-^ HUVECs in uremic serum compared to GSTM^+/-^ HUVECs in control serum.

According to proteome array analysis, ICAM-1 and VCAM-1 expression was elevated in HUVECs silenced for the GSTM1 gene, although these did not achieve the set 2-fold threshold limit. Given that we previously demonstrated ICAM-1 and VCAM-1 as key biomarkers of cardiovascular survival in ESRD patients [17], we investigated the changes in these proteins separately using Western blotting (Figures 5(a) and 5(b)). The incubation of HUVECs with uremic serum led to a significant increase in ICAM-1 expression in GSTM^+/-^ HUVECs compared to control serum conditions (*p* < 0.05). GSTM1 knockdown led to the increase in ICAM-1 expression in HUVECs incubated in control serum, which was more pronounced in HUVECs incubated in uremic serum (*p* < 0.05). Similarly, HUVECs silenced for the GSTM1 gene had higher expression of VCAM-1 when incubated in control serum, as well as in uremic serum treatment when compared to HUVECs with normal levels of GSTM1; however, this was not statistically significant.

**Figure 6:**
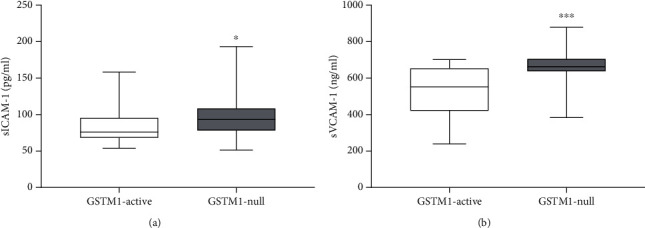
The influence of GSTM1 deletion on sICAM and sVCAM plasma concentration in ESRD patients. A cohort of ESRD patients was genotyped for GSTM1 gene by multiplex PCR. sICAM-1 and sVCAM concentrations were determined in plasma of ESRD patients by ELISA. Results are presented as the median with interquartile range. ^∗^*p* < 0.05, ^∗∗∗^*p* < 0.001.

Using clinical plasma samples from patients with ESRD, we compared the concentrations of soluble adhesion molecules, sICAM-1 and sVCAM-1, between patients with *GTSM1-null* and *GSTM1-active* genotypes (Figures 6(a) and 6(b), respectively). These results present refined analyses of the recent findings on oxidative stress and endothelial dysfunction biomarkers that can predict survival in ESRD patients [17]. As hypothesized, sICAM-1 levels were 24% higher in patients with *GSTM1-null* genotype compared to the *GSTM1-active* genotype (93.28 (78.34-108.16) pg/ml and 75.34 (68.32-95.4) pg/ml, respectively, *p* < 0.05). Similarly, patients with *GSTM1-null* genotype had 24% higher levels of sVCAM-1 levels (662.38 (637.98-704.38) ng/ml), in comparison to ESRD patients with *GSTM1-active* genotype (532.51 (420.78-653.24) ng/ml, *p* < 0.001).

## 5. Discussion

In this study, we showed that uremic serum caused redox imbalance independently of the GSTM1 knockdown, as well as an alteration in the expression of a series of inflammatory cytokines. Moreover, the reduction in GSTM1 in HUVECs led to an increase in MCP-1 expression. In addition, GSTM1 knockdown induced *in vitro* upregulation of cell adhesion molecules and markers of endothelial dysfunction, ICAM-1 and VCAM-1, which has also been confirmed in the clinical settings using plasma samples from patients with ESRD.

GSTM1 belongs to the detoxifying group of enzymes able to remove numerous reactive compounds from the body [24, 25]. Patients on haemodialysis homozygous for GSTM1 deletion are at higher overall and cardiovascular mortality risk [16, 17], most likely due to higher oxidative burden [8]. Given the pivotal role that oxidative stress plays in the development of endothelial dysfunction, we postulated that the lack of GSTM1 activity contributes to the formation of ROS and oxidative damage. To further elucidate the effects of the GSTM1 reduction on oxidative stress in uremic conditions, in this study, we performed a series of HUVEC-based *in vitro* experiments following GSTM1 knockdown. The results presented herewith show that uremic serum caused a decrease in both SOD and GPX antioxidant enzyme activities, which was accompanied by an increase of MDA levels. These results are in line with the *in vitro* study of Chen et al., as well as well-documented increase of MDA levels and downregulated extracellular antioxidant capacity in the clinical settings [26–28]. Taken together, our results provide further evidence that endothelial cells contribute to systemic oxidative stress in uremia. This effect on oxidative stress in HUVECs exposed to uremic conditions appears independent of GSTM1 levels.

Regarding the expression of cytokines, incubation in uremic serum resulted in markedly higher expression of RBP4, RANTES, CRP, and angiogenin, while the expression of Dkk-1 and PF4 was suppressed. These changes should be interpreted in the context of complex pathophysiology of ESRD patients' vasculature, often leading to arteriosclerosis and atherosclerosis. Disturbance of calcium and phosphate homeostasis together with uremic toxins plays a key role in arteriosclerosis and contributes to accelerated calcifications of arteries in CKD patients. One of the proteins involved in mineralization or calcification of arterial walls is Dkk-1 [29]. Dkk-1 is a well-established protein associated with impaired osteoblast activation and bone loss. Up to date, several approaches to study Dkk-1 suggest its protective role in arterial calcifications. Interestingly, negative association between circulating Dkk-1 and arterial stiffness was found in predialysis CKD patients [30]. In the present study, we have shown, for the first time, that components of the uremic serum alter the expression of Dkk-1 in HUVECs. Our results are in line with those of other authors that report a negative association between circulating Dkk-1 and arterial calcified plaques in type 2 diabetes mellitus [31]. Although our findings should be confirmed in larger clinical studies, these results likely suggest that Dkk-1 could be a potential therapeutic target in ESRD.

Atherosclerosis is primarily a disorder of the intima of medium diameter arteries, characterized by plaque formation, narrowing, and occlusion of the blood vessels. It is likely that the mechanism of atherosclerosis in ESRD patients includes the same events as in the rest of the population without CKD. However, the rate of progression of atherosclerosis as well as the degree of oxidative modifications, expression of adhesion molecules, formation of foam cells, and proliferation of smooth muscle cells is more pronounced in CKD patients. Results of our study showed that uremic serum exerted changes in the expression of several molecules involved in the atherosclerotic processes in HUVECs. These proteins have been shown to stimulate either cell proliferation (angiogenin) or monocyte adhesion (ICAM-1, RANTES, and PF4). Angiogenin induces angiogenesis by activating endothelial and vascular smooth muscle cells [32]. According to our results, uremic serum led to an increase in endothelial angiogenin expression. It has been shown before that angiogenin levels increase significantly with CKD progression [33]. Moreover, high angiogenin levels have been linked with peripheral occlusive arterial disease and acute coronary syndrome [34, 35]. Therefore, our results indicate that the endothelium might contribute to accelerated atherosclerosis in uremic conditions by upregulating angiogenin expression. During the early stages of atherosclerosis, stimulation of endothelial cells results in the secretion of adhesion molecules, leading to the recruitment of leukocytes to the vascular wall. ICAM-1 is constitutively expressed, whereas VCAM-1 is weakly expressed by resting endothelium [36]. Both molecules are upregulated by inflammatory stimuli. Our western blot analysis showed increased expression of ICAM-1, but not VCAM-1, in HUVECs incubated in uremic serum. Our results are in line with the study of Tumur et al. who found that one of the uremic toxins, indoxyl sulfate, significantly increased ICAM-1 expression in HUVECs, while this effect on VCAM-1 was slower [36]. In our experimental design, the incubation in uremic serum lasted only 6 h, which may not be a sufficient time for endothelial response in terms of VCAM-1 expression. Previous reports showed that CKD patients have elevated levels of ICAM-1 and VCAM-1 adhesion molecules [37]. The possible explanation might be that elevated levels of VCAM-1 in patients with CKD are influenced not only by the endothelium but also by the other sources such as monocytes and macrophages. An additional adhesion molecule, whose expression was increased upon incubation in uremic serum in our study, was RANTES. RANTES mediates transmigration and arrest of monocytes onto activated endothelium. To our knowledge, this is the first report on upregulated RANTES expression in uremic conditions *in vitro*. RANTES is a well-established mediator of atherogenic processes [38]. Moreover, the importance of RANTES in renal disease was established in a study of renal transplants undergoing rejection [39], since rejecting grafts exhibited large amounts of RANTES bound to the vascular endothelium. In our study, RANTES was expressed in HUVECs exposed to uremic serum, while the HUVECs incubated in control serum did not have visually detectable expression of this protein. In our study, the only adhesion molecule with reduced expression in uremic serum was PF4. Given the fact that PF4 inhibits progenitor cell proliferation and angiogenesis [40], its decreased expression in HUVECs might result in atherosclerosis promotion and therefore seems biologically plausible.

Additionally, in our study, HUVECs incubated in uremic serum showed increased expression of two acute-phase reactants, RBP4 and CRP. RBP4 is a plasma protein, which is mainly secreted by the liver and adipose tissue, and is known to transport retinol in the blood [41]. It has been shown that RBP4 elevation induces inflammation in primary human retinal microvascular endothelial cells (HRECs) and HUVECs by increasing the expression of proinflammatory cytokines, chemokines, and adhesion molecules [42]. RBP4 levels have also demonstrated positive association with the degree of carotid intima media thickness [43]. Moreover, RBP4 levels have been reported to be elevated in kidney disorders in late stages [44]. Notably, Frey et al. showed that the relative amount of RBP4 isoforms was increased in CKD patients in comparison to controls [45]. In relation to other well-established marker of inflammation, CRP was over 2-fold overexpressed in HUVECs incubated in uremic serum compared to control serum. HUVECs express CRP mRNA and protein constitutively, revealing that vascular cells are another source of CRP production [46]. In addition to being a marker of inflammation, a growing body of evidence suggests that CRP may directly participate in the development of atherosclerotic vascular disease. Therefore, elevated CRP levels have emerged as one of the most powerful independent predictors of cardiovascular disease. Our study showed for the first time that CRP expression might increase in endothelial cells upon exposure to uremic serum.

It is tempting to speculate that regulatory and executive adhesion molecule response to uremic serum could be a consequence of activation of the overlapping signaling pathway. Namely, the promoters of ICAM-1, RANTES, RBP4, and CRP genes contain binding sites for the transcription factor NF-*κ*B [36, 47–49]. It is well established that uremic toxins promote oxidative stress which activates the NF-*κ*B signaling pathway in HUVECs [50–53]. Given that uremic serum led to redox imbalance in HUVECs in our study, induction of the NF-*κ*B signaling pathway might be one of the possible mechanisms potentially explaining the upregulation of aforementioned adhesion molecules in HUVECs.

Silencing of GSTM1 in HUVECs led to an increased expression of endothelial adhesion molecules including MCP-1 and ICAM-1 and a possible trend in VCAM-1 overexpression. Notably, the MCP-1 expression increased over 2-fold in response to GSTM1 knockdown in HUVECs incubated in both control and uremic serum. With respect to the role of MCP-1 in attracting monocytes to the site of vascular injury, our results provide one of the mechanistic clues for higher risk of cardiovascular diseases in subjects lacking GSTM1 (*GSTM1-null* genotype), which is even more pronounced in uremic milieu in ESRD patients. Our results are in line with the very recent report of Gigliotti et al., which showed that GSTM1 knockout mice had a significant increase in renal expression of MCP-1 [54]. MCP-1 is also an important factor in the pathogenesis and progression of renal failure [55]. Higher urinary MCP-1 concentrations were found in CKD patients and correlated with kidney damage. Although the precise mechanism of GSTM1-mediated regulation of MCP-1 remains elusive, it is important to note that GSTM1 has a functional noncatalytic domain that inhibits activation of the apoptosis signaling-regulating kinase 1 (ASK1)-p38 signaling pathway [56]. Terada et al. reported that ASK1 directly regulates MCP-1 expression [57]. Moreover, p38 MAPK-mediated regulation of MCP-1 expression has also been confirmed in HUVECs [58]. Therefore, it is reasonable to assume that the lack of GSTM1 protein in GSTM1^+/-^ HUVECs results in higher expression of MCP-1 due to the lack of ASK1 inhibition. Similarly, it is likely that another two upregulated proteins, ICAM-1 and VCAM-1, in response to GSTM1 knockdown might be associated with the ASK1 signaling pathway [59]. This should be explored in future studies.

## 6. Conclusions

The results of the present study show that uremic serum caused redox imbalance characterized by enhanced lipid peroxidation and decreased antioxidant enzyme activities, independently of the GSTM1 knockdown. In response to uremic injury, HUVECs exhibited changes in the expression of a series of cytokines involved in either arteriosclerosis and/or atherosclerosis, some of which might be relevant as therapy targets. Markers of endothelial dysfunction, ICAM-1 and VCAM-1, were both increased in ESRD patients lacking GSTM1, while ICAM-1 was upregulated in endothelial cells with a low level of GSTM1 exposed to uremic serum, further strengthening their potential biomarker role as predictors of CVD in ESRD patients. Interestingly, our study describes a novel function of endothelial GSTM1 in the regulation of monocyte migration and adhesion, through its role in the upregulation of MCP-1. Future studies confirming and expanding on these findings with the inclusion of functional assays of cell adhesion migration, and invasion would strengthen of our results.

Based on these results, it may be concluded that incubation of endothelial cells in uremic serum induces redox imbalance accompanied with altered expression in a series of cytokines involved in arteriosclerosis and atherosclerosis pathogenesis. The association of GSTM1 downregulation with the altered expression of adhesion molecules might be at least partly responsible for increased susceptibility of ESRD patients to CVD.

## Figures and Tables

**Table 1:** Array key.

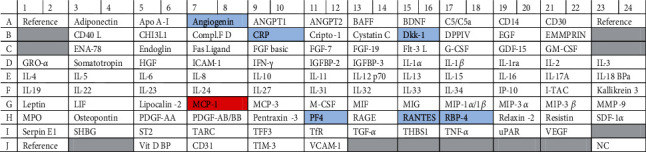

## Data Availability

The data used to support the findings of this study are available from the corresponding authors upon request.

## Supplementary Materials

Supplementary 1 Figure 1S: the viability of HUVECs. HUVECs (*n* = 6) were incubated in growth medium; 10%, 20%, and 30% control and uremic serum-containing media for 4 h and 6 h. Cell viability was determined by the MTS test.

Supplementary 2 Figure 2S: GSTM1 knockdown in HUVECs. GSTM1 siRNA successfully knocked down GSTM1 expression in HUVECs by ~90% when compared to the control. HUVECs were treated with 100 nM siRNA. 96 h posttransfection, the GSTM1 reduction was confirmed by Western blot (representative pictures inset). Results are presented as the mean ± SD, *n* = 3, ^∗∗∗^*p* < 0.001.

Supplementary 3 Figure 3S: expression of a panel of cytokines determined by Proteome Profiler Human XL Cytokine Array. HUVECs (*n* = 3/group, pooled), transfected with GSTM1 siRNA and GSTM1^+/+^ HUVECs were incubated in 30% control or uremic serum-containing media for 6 h. Heatmap represents pixel densities of spots normalized by respective reference spots.
