# Supplementary material for: GSTM1 Modulates Expression of Endothelial Adhesion Molecules in Uremic Milieu
Source: Oxid Med Cell Longev. 2021 Jan 25;2021:6678924. doi: 10.1155/2021/6678924 (PMC7860968; doi:10.1155/2021/6678924)
Supplement: Supplementary 1 — Figure 1S: the viability of HUVECs. HUVECs (n = 6) were incubated in growth medium; 10%, 20%, and 30% control and uremic serum-containing media for 4 h and 6 h. Cell viability was determined by the MTS test. [file 6678924.f1.docx]

A)

B)

**Figure 1S. The viability of HUVECs.** HUVECs (n=6) were incubated in medium; 10%, 20%, 30% control and uremic serum for: A) 4 h and B) 6 h. Cell viability was determined by MTS test.
