# Supplementary material for: GSTM1 Modulates Expression of Endothelial Adhesion Molecules in Uremic Milieu
Source: Oxid Med Cell Longev. 2021 Jan 25;2021:6678924. doi: 10.1155/2021/6678924 (PMC7860968; doi:10.1155/2021/6678924)
Supplement: Supplementary 2 — Figure 2S: GSTM1 knockdown in HUVECs. GSTM1 siRNA successfully knocked down GSTM1 expression in HUVECs by ~90% when compared to the control. HUVECs were treated with 100 nM siRNA. 96 h posttransfection, the GSTM1 reduction was confirmed by Western blot (representative pictures inset). Results are presented as the mean ± SD, n = 3, ∗∗∗p < 0.001. [file 6678924.f2.docx]

**GSTM1**

**(25 kDa)**

**β-actin**

**(42 kDa)**

**GSTM1^+/-^  GSTM1^+/+^**


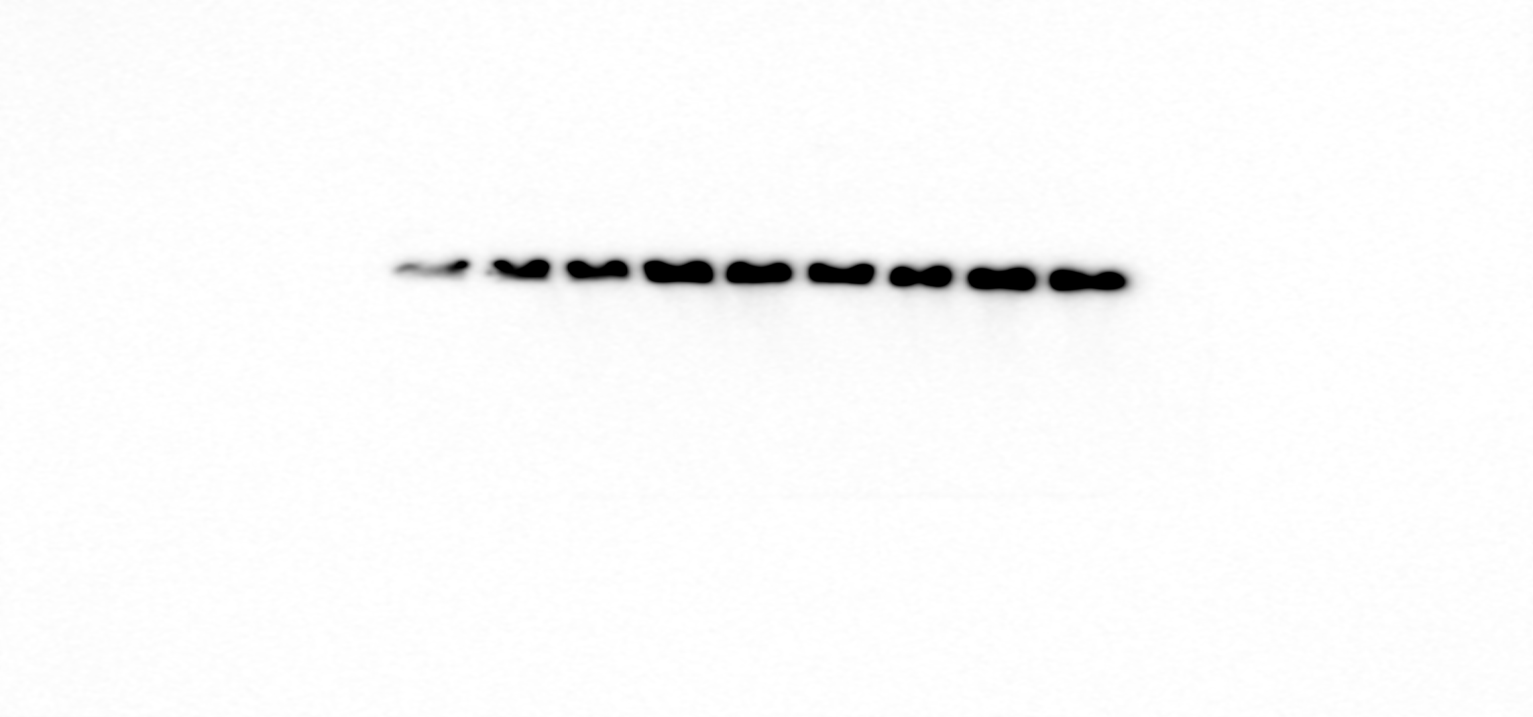

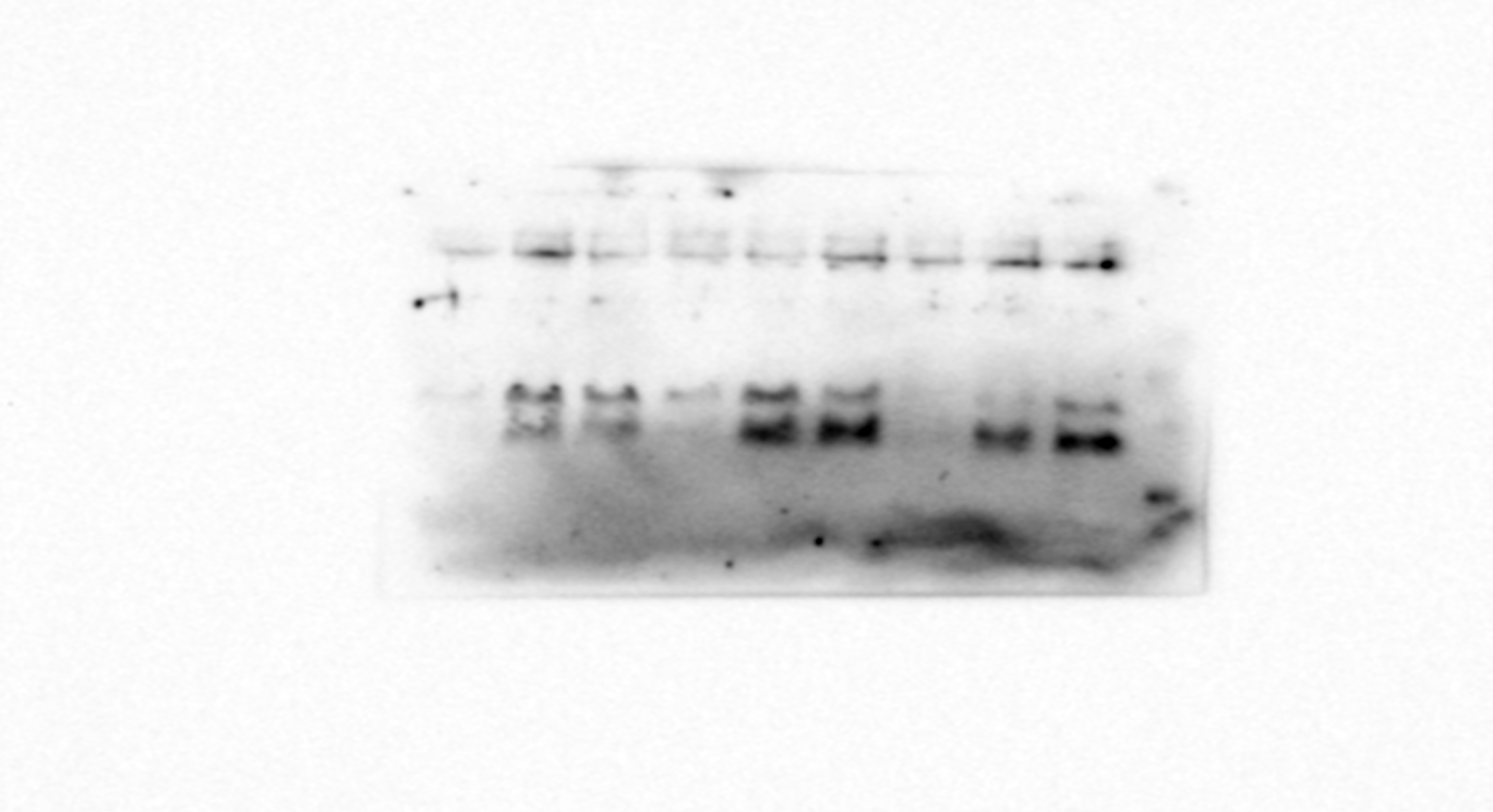


** *

**Figure 2S*.* GSTM1 knockdown in HUVECs.** GSTM1 siRNA successfully knocked down GSTM1 expression in HUVECs by ~90% when compared to control. HUVECs were treated with 100 nM siRNA. 96 h post transfection, the GSTM1 reduction was confirmed by Western blot (representative pictures inset). Results are presented as mean ± SD, n=3, ***p<0.001
