# Supplementary material for: GSTM1 Modulates Expression of Endothelial Adhesion Molecules in Uremic Milieu
Source: Oxid Med Cell Longev. 2021 Jan 25;2021:6678924. doi: 10.1155/2021/6678924 (PMC7860968; doi:10.1155/2021/6678924)
Supplement: Supplementary 3 — Figure 3S: expression of a panel of cytokines determined by Proteome Profiler Human XL Cytokine Array. HUVECs (n = 3/group, pooled), transfected with GSTM1 siRNA and GSTM1+/+ HUVECs were incubated in 30% control or uremic serum-containing media for 6 h. Heatmap represents pixel densities of spots normalized by respective reference spots. [file 6678924.f3.docx]

**Figure 3S. Expression of a panel of cytokines determined by Proteome Profiler Human XL Cytokine Array.** HUVECs (n=3/group, pooled), transfected with GSTM1 siRNA and GSTM1^+/+^ HUVECs were incubated in 30% control or uremic serum containing media for 6 h**.** Heatmap represents pixel densities of spots normalized by respective reference spots.
